# Perceiving Speaker’s Certainty: The Interaction Among Subjectivity of Statement, Evidential Markers, and Evidence Strength

**DOI:** 10.3390/bs15070912

**Published:** 2025-07-04

**Authors:** Qiang Fang, Yinmei Li

**Affiliations:** 1 School of Foreign Studies, Yanshan University, No. 438 West Hebei Avenue, Qinhuangdao 066004, China; 2 School of Humanities and Law, North China University of Technology, No. 5 Jinyuanzhuang Road, Shijingshan District, Beijing 100144, China; liyinmei@ncut.edu.cn

**Keywords:** perceived speaker’s certainty, evidential markers, subjectivity, objectivity, evidence strength

## Abstract

Evidentiality is a linguistic category whose primary meaning is the source of information, which is generally divided into firsthand perception, hearsay, and inference. Evidential markers are the linguistic devices that indicate information sources. While considerable studies have revealed that evidential markers have an effect on the perceived speaker’s certainty, no comparison on such an effect has been carried out in subjective and objective sentences. Moreover, with evidential markers and evidence strength both influencing the speaker’s certainty, their relationship when used in collocation has not been investigated. This article examined, in two experiments, the intertwinement of inferential markers, subjectivity, and evidence strength in affecting the perceived speaker’s certainty in Chinese. Participants were asked to judge the perceived speaker’s certainty in sentences with and without inferential markers and also in sentences with different degrees of evidence strength in subjective and objective conditions. Our results revealed that (i) subjective evaluations are conceived with a lower degree of the speaker’s certainty than objective sentences; (ii) Chinese evidential markers change the perceived speaker’s certainty in subjective and objective sentences; and (iii) evidence strength plays a role in subjective evaluations but not in objective sentences. Our results show the participants reasoning the subjective evaluation themselves based on the evidence, but adopting the speaker’s evaluation on the objective situations. Overall, our study supported that inferential markers significantly lowered the perceived speaker’s certainty, and reported the different effects of evidence strength in objective sentences and subjective evaluations.

## 1. Introduction

Degrees of certainty pertain to our lives, whenever we make judgments or utter linguistic expressions. Theoretical investigations into certainty have a long history, often drawing on linguistic data from diverse languages, such as those presented in Faller (2002) and Matsui et al. (2016). Various experiments have been conducted concerning the certainty of linguistic expressions, for example, whether explicit claims of certainty are more persuasive than plain, unqualified statements (Teigen & Juanchich, 2024). Certainty is also experimentally tested to be relative to the evidence strength and presentation method (Martire et al., 2013), evidential and modal expressions (Tosun & Vaid, 2018), evidential markers (Arslan, 2020), contour and epistemic modality (Gravano et al., 2009), and inference and guess (Pillow, 2002). Nevertheless, the relations between the speaker’s certainty and various other parameters remain under-explored, such as speaker–hearer familiarity, and the subjectivity of the speech content. It remains unclear how the subjectivity of the speech content, inferential markers, and evidence strength interact and influence the perceived speaker’s certainty in the inferential process. We argue that subjective evaluations and objective sentences do not perceive the same degree of the speaker’s certainty and evidence strength has dissimilar influences in the two conditions. To the best of our knowledge, a comparison of subjective and objective conditions of the speech content has not been attempted previously. Moreover, the participants are found to rely on the evidence to form their own subjective evaluations, but they adopt the speaker’s evaluation on the objective situations. We contribute to the previous studies by providing evidence that adding an evidential marker to a sentence does not necessarily lower its perceived speaker’s certainty, with evidence strength as one of the possible override factors.

### 1.1. Subjective/Objective Statements and Speaker’s Certainty

The word ‘objective’ means expressing or dealing with facts or conditions as perceived without distortion by personal feelings, prejudices, or interpretations (Merriam-Webster Dictionary). Subjectivity means that language use inevitably embodies the speaker himself, his perspective, attitude, and emotions (Lyons, 1977, p. 739). The distinction between subjective evaluations and objective situations lies in their verifiability; whereas objective sentences depicting objective situations are open to verification by others, subjective evaluations are inherently personal and are verifiable solely by the individual expressing them. The difference between objective sentences and subjective evaluations can be illustrated by examples (1) and (2).
(1) 卧室 门 开 了。wòshì mén kāi lebedroom door open -edThe door of the bedroom has opened.(2) 这道 菜 很 好吃。zhèdào cài hěn hǎochīthis dish very deliciousThis dish is very delicious.

In (1), ‘the door has opened’ describes an objective situation that can be verified by the hearer, and the norm is context-based as it pertains to external, factual reality. In (2), ‘this dish is very delicious’ constitutes an epistemic evaluation grounded in the speaker’s perception. Without tasting the dish themselves, hearers are unable to verify this assertion. Epistemic evaluations are those which mirror the speaker’s own attitudes and are widely recognized as subjective by the three approaches to subjectivity proposed by Lyons (1977), Traugott (1995), and Langacker (1990), as highlighted in Nuyts (2012) (cited from Sanders and Spooren (2015)). Additionally, the degree intensifier *hen* (very) further accentuates the speaker’s perspective, rendering Example (2) paradigmatically subjective. It is worth noting that the closely related dimension of “intersubjectivity”, which signifies the speaker’s consideration of the addressee’s self-image (Traugott, 2010), is not delved into in this context. This is because our analysis is confined to casual conversations among family members and friends.

**Figure 1:**
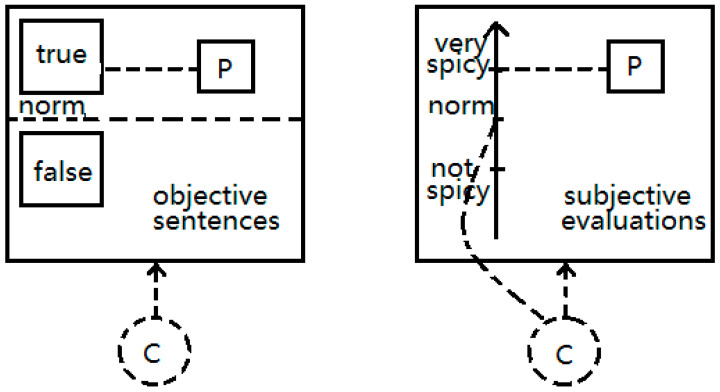
Cognitive schemas of objective sentences and subjective evaluations. (P: proposition, C: conceptualizer; e.g., speaker, hearer, or audience of the conversation).

In the framework of Cognitive Grammar (Langacker, 2008) as shown in Figure 1, the norm of an objective sentence is grounded in the context, while the norm of a subjective evaluation is situated in the mind of the conceptualizers who construe the situation. When construing an objective situation (e.g., “The bedroom door has opened.”), the conceptualizer relies on an objective and shared norm. That is, the status of the door being open is an objective fact and is not subject to the conceptualizer’s personal interpretation. When construing a subjective situation (e.g., “The dish is very delicious.”), the conceptualizers employ their own norm to evaluate the situation described; i.e., ‘delicious’ is a subjective norm and may vary among individuals. From the hearer’s perspective, when perceiving an objective sentence, the hearer has the faith that the speaker has the ability to correctly describe the situation. When perceiving a subjective sentence, the hearer recognizes that the speaker may have a distinct evaluation norm, and the speaker’s description is not universally accepted.

Studies have shown that subjective and objective speech contents may display distinct characteristics when paired with evidential markers. In experiments comparing plain sentences and statements with certainty markers (Teigen & Juanchich, 2024), the participants were asked to judge the speaker’s confidence when uttering plain statements, impersonal certainty statements, and personal certainty statements, such as “Jack owns two cars”, “I am certain Jack owns two cars”, and “It is certain Jack owns two cars”. The participants were to judge on a 4-point scale of acceptability (1: Needs checking, 2: Might need checking, 3: Might not need checking, and 4: Does not need checking). In most scenarios, the plain statements were judged to be more trustworthy than those qualified as certain. Scenarios are often perceived as more likely when they are based on objective observations (for instance, measurements and instruments) rather than on subjective judgments (such as memories and intuitions). This suggests that the impact of the inherent subjectivity or objectivity of information on the perception of statement certainty merits further investigation beyond the use of evidential markers.

In another study, Lee and Kaiser (2021) investigated how subjectivity affects the perceived generalizability of opinions. They examined two types of subjective adjectives: predicates of personal taste (PPTs), such as “The cake is tasty”, and non-PPT multidimensional adjectives (MDs), such as “Tom is healthy”, in conjunction with Korean evidentials. Participants were tasked with imagining a scenario where they visited an alien planet and observed the aliens’ conversations, which included either PPTs or MDs. PPTs, requiring direct and personal experience, seem more subjective, unlike MDs, which permit ranking or ordering individuals based on a property and can be negated. Different evidential markers, -*te*- for direct, -*napo*- for inference and -*tay* for hearsay, were added to a baseline statement such as ‘The pulon is tasty’. Participants were asked to judge the generality of ‘The pulon is tasty (PPT)’, after hearing four kinds of evidential sentences (baseline, direct, inference, and hearsay), by answering ‘If we select 100 random aliens that live on this planet, how many of them would have the opinion that the [nonce noun] is [PPT/MD]?’. The results indicated no evidentiality effects with PPTs, whereas, with MDs, evidentiality significantly influenced the perceived generality of opinions.

The source of certainty or uncertainty is argued as attributable to internal (subjective) or to external (objective) factors (Teigen & Juanchich, 2024). Moreover, subjective adjectives requiring varying degrees of direct and first-person experience, behave differently in collocation with Korean evidentials (Lee & Kaiser, 2021). These findings prompt the hypothesis that subjective and objective speech contents may exhibit distinct behaviors when paired with evidential markers.

### 1.2. The Role of Evidentiality in Modulating (Perceived) Speaker’s Certainty

Evidentiality pertains to the source of information for a proposition (e.g., Aikhenvald, 2004, p. 1). Information sources are universally either direct (perceptual) and indirect (reported and inference). It has been demonstrated that evidential markers can diminish the perceived certainty of a speaker’s statements (e.g., Hara et al., 2020; Smirnova, 2021). For instance, in one Bulgarian experiment, Smirnova (2021) asked participants to decide the two protagonists’ certainty about their conflicting inference results solely based on the reactions of the crowd of a football match between teams A and B. One protagonist employed an evidential form for ‘A team scored’ while the other used a non-evidential form for ‘B team scored’. The results revealed that the claim with an evidential marker is perceived as less certain than that without an evidential marker (Smirnova, 2021). These findings suggest that evidential markers do not merely indicate the source of information for a proposition (e.g., Aikhenvald, 2004, p. 1) but also semantically overlap with epistemic modality, which marks the speaker’s degree of certainty and/or the necessity/possibility of the truth of the propositional content (Faller, 2006).

Tosun and Vaid (2018) examined the relationship between the source type and confidence (certainty) of the evidential and modal sentences in Turkish and English. In each language, the stimuli consisting of 80 declarative sentences in active voice, third person singular form, and past tense were presented in eight versions each with a set of ten sentences. Four evidential and four modal forms of the sentences were added to each set of the sentences. Participants were asked to assess the confidence of the sentences based on the content of the sentences themselves. The ‘not enough information’ responses constitute up to 10% for some cases and are hardly reported in other cases. After excluding the ‘not enough information’ cases, the participants were asked to judge the sentence type and the confidence level by choosing 3 (extremely confident), 2 (quite confident), 1 (somewhat confident), or 0 (not at all confident). The relationship between the sentence (evidential) types and the confidence is calculated in the evidential condition, as well as the relationship between the modal types and the confidence in the modal condition. The results indicated that evidence types have a significant main effect on confidence.

Arslan (2020) explored the impact of epistemic uncertainty on the processing of grammatical evidentiality in Turkish through four experiments. In Experiment 1, participants were asked to assess the acceptability of the verb evidentiality in the given contexts. The mismatches with an indirect evidential form in the witnessed context or those with a directed evidential form in the reported context were observed with a high unacceptability rate. In the acceptability judgment tasks in Experiment 2, the use of indirect evidentiality is deemed less acceptable in first-hand witnessed contexts, irrespective of whether the speaker is certain about the witness. The acceptability judgment tasks in Experiment 3 suggest Turkish speakers judge contexts expressing one’s own witnessed information as less acceptable when paired with a low certainty. In experiment 4, participants were asked to complete a story with a sentence after reading the context and critical sentences, selecting either a direct evidential, an indirect evidential, or an assumption marker. The results showed that the high certainty condition favored a direct evidential, while the low certainty condition favored the assumption marker. The choice of indirect evidentiality did not exhibit a significant difference between the high and low certainty conditions.

Fishman and Degen (2023) addressed the debate on whether the uncertainty in inference is attributed to extra-linguistic reasoning about the evidence directness or to pragmatic reasoning about alternative utterances through the two experiments. In Experiment 1, participants were presented with utterances and had to rate the speaker’s certainty under the influence of evidence directness. Informed of the discourse context, each participant was to judge the speaker’s certainty of 20 sentences by adjusting a slider with endpoints labeled 1 (absolutely sure) to 0 (not sure at all) to indicate the speaker’s certainty. The 20 stimuli consist of 5 bare utterances, 5 utterances with ‘looks’, and 10 fillers. Each stimulus was paired with one of 10 adjectives that varied in visual strength. No main effects of visual strength or squared visual strength were found. However, a significant interaction between the utterance type and squared visual strength was observed. In Experiment 2, participants were placed in the speaker’s role and had to decide which possible utterances to use in specific situations. This was carried out to examine the impact of evidence directness and perceptibility conditions on their choices. Three perception verbs—*look*, *sound*, or *feel*—were used, matching the source based on the context information. The contexts varied in terms of perceptual qualities, such as good visual evidence and bad auditory evidence. Each utterance contained one of the adjectives with different perceptual strengths. The mixed-effects logistic regression analysis revealed no main effect of perceptibility conditions or perceptual strength, but a significant effect of squared perceptual strength was observed. Specifically, evidential devices with extremely low or high perceptual strength were less likely to be used in contexts with good perceptibility conditions. The results suggest that strong evidence leads to a high speaker certainty, with more bare utterances being adopted, while weaker evidence, which is less direct or reliable, leads to the increased use of evidential devices. The findings support an account that combines both extra-linguistic reasoning about evidence directness and pragmatic reasoning about alternative utterances.

It is worth noting that most previous studies examined the effect of evidential markers in objective sentences that depict factual situations (Tosun & Vaid, 2018; Arslan, 2020; Smirnova, 2021; Teigen & Juanchich, 2024), with very few adopting subjective sentences (Lee & Kaiser, 2021; Fishman & Degen, 2023). Subjective evaluations are inherently inferential and speaker-centric, marking a significant contrast with the nature of objective situations. The impact of subjective versus objective statements on the perceived certainty of the speaker, particularly when employing inferential markers, remains an area ripe for further exploration.

### 1.3. Chinese Evidential Markers

Chinese uses lexical devices to indicate information sources, which can be divided into the direct and indirect forms, with the indirect form being further divided into hearsay and inference (Zhu, 2006; Yue, 2014). Evidentiality in Chinese is coded by different kinds of lexical categories, such as verbs, modal particles, adverbs, adjective, and nouns (Zhu, 2006).
(3) 我 看见/ 想/ 听说 外面 在 下雨。wǒ kànjiàn/ xiăng/ tīngshuō wàimiàn zài xiàyǔI see/think/hear outside -ing rainI see/think/hear it is raining outside.(4) 依 我 看/ 推断/ 听说， 那 不 是 真的。yī wǒ kàn/ tuīduàn/ tīngshuō, nà bù shì zhēndeaccording I see/infer/hear, that not be trueAccording to me/based on my inference/from what I hear, that is not true.

As illustrated in (3) and (4), lexical evidential markers of direct and indirect evidentiality in Chinese exhibit flexible positioning, either within the sentence as the predicate or separated at the beginning of the sentence. Languages without grammatical evidential markers are of great importance for understanding evidentiality as a semantic category.

In languages with grammaticalized evidential markers, subjective sentences are coded with evidential markers by default, and subjective sentences without evidential markers are typically ungrammatical. Languages using lexical evidential markers like English and Chinese provide minimal pairs for subjective sentences with and without an evidential marker. For example, by comparing ‘I think it is a good idea’ vs. ‘It is a good idea’, the function of ‘I think’ can be identified.

There has been a theoretical debate regarding whether evidentiality can be separated from epistemic modality (reliance of information source or speaker’s commitment) (e.g., the “disjunction view”, De Haan, 1999; Aikhenvald, 2004; Cornillie, 2009; the “inclusion view”, Chafe, 1986; Plungian, 2001; Bybee & Pagliuca, 1985; Willett, 1988; the “overlapping view”, Palmer, 1986; Du Bois, 2007; González et al., 2017). In the Chinese literature, some Chinese researchers (e.g., Fang, 2006; Du, 2015) do not make a strict distinction between the information source and the speaker’s commitment, certainty, or reliability associated with that source, indicating an overlap view between evidentiality and epistemic modality. The speaker’s commitment or certainty is considered an intrinsic aspect of the meaning conveyed by evidentiality, suggesting an overlap relationship between evidentiality and (epistemic) modality. On the other hand, the commitment is viewed as a pragmatic implicature of evidential markers but not their core meaning, supporting the ‘disjunction’ view between evidentiality and epistemic modality. The two categories share some similar pragmatic functions but with no semantic overlap (e.g., Gan & Yu, 2024; Yue, 2014). It is worth noting that both views do not strictly conflict with each other. They share a general agreement that Chinese evidential markers denote a modal function. For example, the use of *wo guji* ‘I reckon’ could soften the speaker’s evaluation of the statement described and result in a comparable perceived speaker’s certainty in (5) and (6).
(5) 我 估计 卧室 门 开 了。wǒ gūjì wòshì mén kāi leI reckon bedroom door open -edI reckon the door of the bedroom has opened.(6) 我 估计 这道 菜 很 好吃wǒ gūjì zhèdào cài hěn hàochīI reckon this dish very deliciousI reckon this dish is very delicious.

The question remains, however, as to whether the softening effect in objective sentences like (5) is the same as that in subjective sentences like (6). To date, no experimental evidence has been provided to either support or refute the scope of evidentiality in Chinese.

### 1.4. Evidence Strength and Speaker’s Certainty

The influence of context and the potency of evidence are pivotal in shaping probability assessments, which, in turn, significantly relate to the perceived speaker’s certainty. In science, high-quality or strong evidence is associated with a substantial change in their belief in the truth of a claim, while weak evidence corresponds to a minor change (Government of Canada 2022, https://science.gc.ca/site/science/en/office-chief-science-advisor/scientific-integrity/resources-and-tools-effective-science-advice/science-shorts-3-strength-scientific-evidence, accessed on 1 January 2025). Strong evidence leads to a strong speaker’s certainty, but the impact of weak evidence seems more complicated. A counterintuitive “weak evidence effect” is reported when participants who received weak positive evidence assessed the outcomes of public policy initiatives as less probable compared to those given no evidence, despite the evidence being deemed supportive when evaluated in isolation (Fernbach et al., 2011; Barnett et al., 2022). In Fernbach et al. (2011), participants were asked to judge the possibility of a conditional question and a marginal question from 0 (impossible) to 100 (definite). The conditional question is made by adding a weak cause (evidence) to the marginal one, as shown in (7) and (8), respectively.
(7) Conditional: The democratic government of Afghanistan is embroiled in a protracted conflict with Taliban insurgents. The European Union recently pledged 9000 troops to provide added security in population centers. How likely is it that Afghanistan will have a stable government in 5 years?(8) Marginal: The democratic government of Afghanistan is embroiled in a protracted conflict with Taliban insurgents. How likely is it that Afghanistan will have a stable government in 5 years?

The results show that the possibility of the result ‘*Afghanistan will have a stable government in 5 years*’ viewed in conditional judgments are much lower than it is viewed in the marginal, though the weak evidence ‘*The European Union recently pledged 9000 troops to provide added security in population centers.*’ separately is judged to raise the possibility.

Evidence strength is argued to be a key factor influencing the speaker’s certainty (Degen et al., 2019). In Experiment 1 of Degen et al. (2019), participants were asked to rate the evidence strength (probability) of four sets of propositions, with four kinds of evidence types, direct, inferential, reported, and wishful, evenly distributed in each set. The evidence strength is found to be systematically predicted by the evidence type, with direct evidence and reported evidence being stronger than inferential or wishful evidence in both English and German. In Experiment 2 and 3, participants were tasked with deciding which evidence type and evidence strength (among bare, direct, inferential, reported, or wishful) are the best choice for propositions with different degrees of modal meaning (evidence strength) (e.g., “It’s raining”, “It must be raining”, “It’s probably raining”, and “It might be raining”). Evidence type and evidence strength are found to be closely correlated with the speaker’s certainty. Although evidentials are generally accepted with an influence on the perceived speaker’s certainty by experiments with cross-linguistic data (e.g., Degen et al., 2019; Smirnova, 2021), the interplay between evidence strength (the cause–effect relation based on evidence presented within the proposition) and evidential markers (inferential markers) appears to be insufficiently explored by experiments.

In conclusion, two research gaps are found: (1) with considerable studies revealing that evidential markers have an effect on perceived speaker’s certainty, no comparison on such an effect has been carried out in subjective and objective sentences; and (2) with evidential markers and evidence strength both influencing speaker’s certainty, their relationship when used in collocation has not been investigated. To fill these gaps, the present study aims to investigate how the subjectivity of a statement interacts with evidential markers and evidence strength in affecting the perception of a speaker’s certainty.

### 1.5. The Present Study

We conduct two experiments on the influence of inferential markers on the perceived speaker’s certainty in a subjective/objective sentence in contexts with or without evidence.

In Experiment 1, we will manipulate the subjectivity of the statement (subjective vs. objective) and the use of evidential markers (with marker vs. bare). We will ask participants to read a context containing critical sentences and rate how certain they think the speaker is. We expect the bare subjective evaluations to result in lower perceived speaker certainty than bare objective statements. Additionally, given that Chinese evidential markers are considered to encode modal meaning, we expect that using evidential markers will soften the speaker’s certainty in both subjective and objective sentences and expect to find no significant difference between the perceived speaker’s certainty in subjective evaluations and objective statements. In Experiment 2, we will include a piece of evidence in the given context. We aim to test how evidence strength interacts with the subjectivity of the statement and the use of evidential markers to affect the perceived speaker’s certainty.

## 2. Experiment 1: Speaker’s Certainty with No Evidence Given in the Context

### 2.1. Methods

#### 2.1.1. Participants

We recruited 244 subjects, either college students or college graduates, 10 of whom were excluded because they did not pass the attention check questions. The remaining subjects comprised 79 males and 155 females (mean age = 23 years, sd = 6 years, age range = 18–52 years).

#### 2.1.2. Materials

##### Selection of Chinese Evidential Markers

We first used ‘*yanju* 言据, *chuanxin* 传信, and evidentiality’ as keywords to search for the journal articles indexed by the China Social Science Citation Index and doctoral dissertations published between 2000 and 2023 (retrieved on 3 March 2023) in the China National Knowledge Infrastructure database (www.cnki.net). In total, 23 papers were found related to evidential expressions in Chinese. Papers with at least one list of evidential expressions are counted as the source of evidential expressions in the current study. The Chinese lexical evidential markers mentioned in these papers were collected, resulting in a list of 238 evidential words (see Supplementary Materials).

We then excluded the non-verbs from the list (i.e., only verbs were selected). Among the remaining verbs (*n* = 100), those that are used as direct evidential markers and reportative evidential markers, and those that can be used in multiple evidential contexts were excluded, resulting in a remaining list of nine evidential verbs: *renwei* ‘think’, *yiwei* ‘think’, *tuice* ‘infer, guess’, *guji* ‘estimate’, *cai*(*ce*) ‘guess’, *tuiduan* ‘infer’, *xiang* ‘think, miss’, *chuaice* ‘think’, and *zuomo* ‘ponder’. These remaining verbs can only be used as inferential markers.

We excluded *xiang* ‘think, miss’ in the next step because it is polysemy. Afterward, the words *chuaice* ‘think’ and *zuomo* ‘ponder’, which cannot lead a subordinate clause (e.g., *wo chuaice ‘I think’/*zhuomo* ‘ponder’ that…), were excluded, resulting in six evidential verbs in the end: *renwei* ‘think’, *yiwei* ‘think’, *tuice* ‘infer, guess’, *guji* ‘estimate’, *cai*(*ce*) ‘guess’, and *tuiduan* ‘infer’. The detailed process of the selection of Chinese evidential verbs is provided in the Supplementary Materials.

##### Construction of Critical Sentences

We initially constructed 28 objective and 28 subjective sentences. Objective sentences followed the structure noun verb-*le* ‘noun has V-ed.’ Subjective sentences followed the structure *zhege*/*nage* noun *hen*/*feichang* adjective ‘this/that noun is very adjective’ where *hen*/*feichang*‘very much’ denotes subjective evaluations. Epistemic evaluation is widely acknowledged across all the approaches to subjectivity. Therefore, we have employed epistemic evaluations as the material for the rating test for subjective sentences. The intensifiers *hen* (very) and *feichang* (meaning: very much, Chinese character 非常), as strong-degree markers, serve to intensify the speaker’s evaluation, functioning as an indicators of subjective evaluation. Additionally, other similar markers exist in Chinese, such as *xiangdang* (meaning: quite, Chinese character: 相当) and *youqi* (especially, Chinese character: 尤其). Among these markers, *hen* and *feichang* are the most frequently used, which are considered to be the most suitable for our research purpose. The objective and subjective sentences were paired with the six Chinese evidential markers, resulting in seven (bare + six markers) sets of objective sentences (total number of 196) and seven sets of subjective sentences (total number of 196).

We asked a separate group of participants (39 males, 110 females, mean age = 21 years, *SD* = 4 years, age range = 18–39 years) to rate the naturalness of the critical sentences in all conditions on a scale of 1–6 (1 represents not natural at all and 6 represents completely natural). Materials used in the real experiments were selected from sentences rated above four. We selected 18 bare objective sentences and 18 bare subjective sentences and their corresponding marked versions (i.e., sentences containing the evidential markers) to make it balanced across conditions. As shown in Appendix A, every 3 of the 18 bare objective sentences were paired with one of the six Chinese evidential markers (i.e., three sentences per marker). The same was ensured for the 18 subjective sentences.

##### Experimental Stimuli

The critical sentences were expanded by adding a situational context to make it sound more natural, as shown in (9)–(12). As shown in (9), no information in the context may result in the speech content described, where whether ‘the door is open’ is irrelevant to the status of ‘you and your dad are sleeping in the living room’. The full list of experimental stimuli is provided in the Supplementary Materials.
(9)Objective-bare 你和爸爸在客厅沙发上睡觉，
nǐhébàbàzàikétīngshāfāshàngshuìjiào
you anddadinliving room sofaonsleep
爸爸对你说：“卧室门开了”。
bàbàduìnǐshuōwòshìménkāile
dadtoyousaybedroomdooropen-ed
‘You and your dad are sleeping on the sofa in the living room. Your dad says to you: *The bedroom door has opened*.’(10)Objective-marked你和爸爸在客厅沙发上睡觉，
nǐhébàbà zàikétīngshāfāshàngshuìjiào
you anddadinliving roomsofaon sleep
爸爸对你说：“我估计卧室门开了”。
bàbàduìnǐshuōwǒgūjìwòshìménkāile
dadtoyousayIreckonbedroomdooropen-ed
‘You and your dad are sleeping on the sofa in the living room. Your dad says to you: *I reckon the bedroom door has opened*.’(11)Subjective-bare你和爸爸今天要在饭店请客人吃饭，
nǐhébàbà jintiānyàozàifàndiànqǐngkérénchīfàn,
youanddadtodaywillinrestauranttreatguesthave meal
你们 来到饭店，爸爸对你说：“这个菜很好吃”。
nǐmén láidàofàndiànbàbàduì nǐshuōzhègècàihěnhǎochī
youcome torestaurantdadtoyousaythisdishverydelicious
‘You and your dad plan to treat some guests in the restaurant today. When you arrive at the restaurant, your dad tells you: *This dish is very delicious*.’(12)Subjective-marked你和爸爸今天要在饭店请客人吃饭， 
nǐhébàbà jintiānyàozàifàndiànqǐngkérénchīfàn,
you anddadtodaywillinrestauranttreatguesthave meal
你们来到饭店，爸爸对你说：“我估计这个 菜
nǐménláidàofàndiàn,bàbàduìnǐshuōwǒgūjìzhègècài
youcome torestaurant dadtoyousayIreckonthis dish
很好吃。”









hěnhǎochī









verydelicious









‘You and your dad plan to treat some guests in the restaurant today. When you arrive at the restaurant, your dad tells you: *I reckon this dish is very delicious*.’

#### 2.1.3. Procedure

The sentences were compiled into four lists in a Latin-square design, each containing 18 objective-bare sentences, 18 objective-marker sentences, 18 subjective-bare sentences, and 18 subjective-marker sentences. In addition, 18 fillers were added to each list. Each participant was randomly assigned to one of the four lists. Participants were asked, ‘How certain do you think the speaker is when he or she said that sentence?’, and instructed to answer on a scale of 1–6 (1 represents not certain at all and 6 represents completely certain).

### 2.2. Results

**Figure 2:**
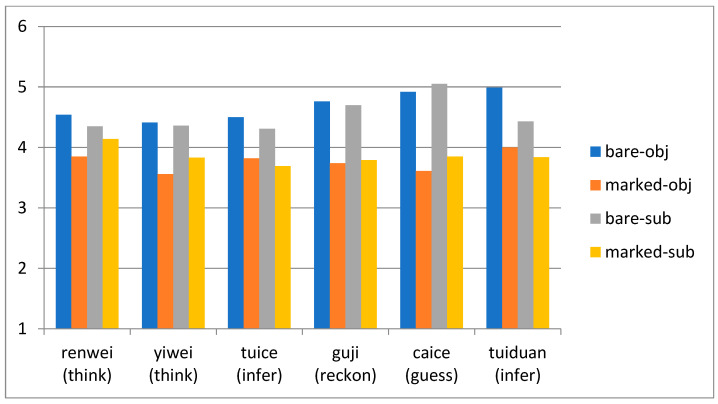
Mean ratings of perceived speaker’s certainty in Experiment 1.

All six inferential markers are found to lower the perceived speaker’s certainty of the speaker in contexts lacking evidence. As one reviewer noted, some markers, like *wo caice* (I guess), overtly diminish the degree of certainty. Other markers, such as *wo renwei* (I think), may not drastically reduce certainty; yet, they still have such an impact, as shown in Figure 2. Moreover, each inferential marker has a more pronounced effect on objective sentences compared to subjective ones. All the examined markers lower the perceived certainty of the speaker, though intra-group differences exist. 

The mean rated speaker certainty was 4.69 (*SD* = 0.35) for objective-bare sentences, 3.76 (*SD* = 0.33) for objective-marker sentences, 4.53 (*SD* = 0.38) for subjective-bare sentences, and 3.85 (*SD* = 0.39) for subjective-marker sentences. Linear mixed-effects models were fitted to the perceived certainty using the lme4 package version 1.1-35.1 (Bates et al., 2015) in R version 4.3.3 (R Core Team, 2024). Subjectivity (sum-coded, subjective −0.5 vs. objective +0.5) and marker (sum-coded, bare −0.5 vs. marker +0.5) were independent variables. Subjects and items were included as random intercepts. Any significant interactions were analyzed with the emmeans package version 1.10.0 (Lenth, 2024). 

We found a main effect of marker (*β* = −0.81, *SE* = 0.03, *t* = −24.36, *p* < 0.001, *CI.lower* = −0.87, *CI.higher* = −0.74), showing that sentences with markers were perceived with a significantly lower speaker certainty than those without markers. In addition, we found a significant interaction between marker and subjectiveness (*β* = −0.24, *SE* = 0.07, *t* = −3.59, *p* < 0.001, *CI.lower* = −0.37, *CI.higher* = −0.11). Post hoc tests showed a significantly smaller effect of marker in subjective sentences (*β* = 0.69, *SE* = 0.05, *t* = 14.60, *p* < 0.001, *CI.lower* = 0.59, *CI.higher* = 0.78) than in objective sentences (*β* = 0.93, *SE* = 0.05, *t* = 19.69, *p* < 0.001, *CI.lower* = 0.83, *CI.higher* = 1.02). The objective-marker sentences and subjective-marker sentences showed no significant differences (*p* > 0.1), but subjective-bare sentences were rated much lower than objective-bare sentences (*β* = −0.15, *SE* = 0.05, *t* = −3.24, *p* < 0.01, *CI.lower* = −0.24, *CI.higher* = −0.06).

We found a main effect of marker (*β* = −0.81, *SE* = 0.03, *t* = −24.36, *p* < 0.001), showing that sentences with markers were perceived with a significantly lower speaker certainty than those without markers. In addition, we found a significant interaction between marker and subjectivity (*β* = −0.24, *SE* = 0.07, *t* = −3.59, *p* < 0.001). Post hoc tests showed a significantly smaller effect of marker in subjective sentences (*β* = 0.69, *SE* = 0.05, *t* = 14.60, *p* < 0.001) than in objective sentences (*β* = 0.93, *SE* = 0.05, *t* = 19.69, *p* < 0.001). The objective-marker sentences and subjective-marker sentences showed no significant differences (*p* > 0.1), but subjective-bare sentences were rated much lower than objective-bare sentences (*β* = −0.15, *SE* = 0.05, *t* = −3.24, *p* < 0.01).

### 2.3. Discussion

The present results were consistent with Smirnova (2021), showing that the inferential markers significantly lower the perceived speaker’s certainty. Additionally, the results indicated marked sentences with inferential markers are significantly lower in terms of the speaker’s certainty perceived than bare sentences, but the decline rate of subjective evaluations is less dramatic than objective sentences. That is to say, inferential markers in Chinese have a stronger influence on objective sentences than subjective evaluations. Such a difference can be attributed to the difference in the subjectivity of the sentences. When evidential markers are incorporated into subjective evaluations, the resulting sentences remain inherently subjective; however, when evidential markers are appended to objective sentences, they transform these statements into subjective ones, reflecting the speaker’s perspective on the objective situation at hand.

## 3. Experiment 2: Perception of Speaker’s Certainty with Provided Evidence

### 3.1. Methods

#### 3.1.1. Participants

We recruited 224 subjects, either college students or college graduates, 12 of whom were excluded because they did not pass the attention check questions. The remaining subjects comprised 99 males and 113 females (mean age = 23 years, *SD* = 6 years, age range = 18–43 years).

#### 3.1.2. Materials

The materials were the same as in Experiment 1 but with a piece of evidence added. Evidence strength is defined as to what extent a speaker would reach a conclusion based on the evidence provided in the context. For example, a piece of evidence, ‘Hearing the sound of the door opening’, was added to objective sentences. The cause–effect relationship between ‘hearing the sound of the door opening’ and ‘saying’, ‘The bedroom door has opened’ is expected to affect participants’ rating of the speaker’s certainty. Similarly, a piece of evidence, “checking the pictures of the dishes”, was added to subjective sentences. Examples of objective and subjective sentences used in Experiment 2 are shown in (13) and (14), respectively. The full list of experimental stimuli is provided in the Supplementary Materials.
(13)Objective sentences with a piece of evidence你和爸爸在客厅沙发上睡觉，
nǐhébàbàzàikétīngshāfāshàngshuìjiào
you anddadstayliving roomsofaonsleep
爸爸听到了开门的声音，对你说：“卧室 bàbàtīngdàolekāiméndeshēngyīnduìnǐshuō wòshìdadhear-edopen door‘ssoundtoyousyabedroom门 开 了”。
mén kāi le
dooropen -ed
‘You and your dad are sleeping on the sofa in the living room. Hearing the sound of the door opening, your dad says to you: *The door of the bedroom has opened*.(14)Subjective statements with a piece of evidence你和爸爸今天要在饭店请客人吃饭，
nǐhébàbàjintiānyàozàifàndiànqǐngkérénchīfàn,
youanddadtodaywillinrestauranttreatguesthave meal
你们来到饭店，爸爸看了一下菜品照片，
nǐménláidàofàndiàn,bàbàkànleyíxiàcàipǐn zhàopiàn
youcome torestaurant,dadsee -ed once dish picture
对你说：“这个 菜很 好吃”。


duìnǐshuō zhègè càihěn hăochī


toyousaythisdishverydelicious


You and your dad plan to treat some guests in the restaurant today. At the restaurant, after checking the pictures of the dishes, your dad tells you: *This dish is very delicious*.

We measured the strength of the provided evidence in each sentence with a separate group of participants (38 males, 15 females, mean age = 21 years, *SD* = 3 years, age range = 18–28 years). This group of participants did not participate in any other experiments in the present study and were asked to read a hypothetical situation like (15) and give a possibility between 0 and 100 (0 represents no possibility and 100 represents inevitability). The statistical analysis will include the strength of the rated evidence in each sentence. The full list of materials and results of the evidence strength test is given in the Supplementary Materials.
(15)假设一个人在 客厅 睡觉，听到 了 开门 的 声音，
jiǎrú yígèrén zài kétīng shuìjiàotīngdàole kāi méndeshēngyīn
suppose a person inrestaurantsleephear -ed open door‘ssound
你 觉得 这个 人 得到 “卧室门开 了” 这个结论
nǐjuédézhègè rén dédàowòshì mén kāi le zhègè jiélùn
youthinkthisperson getbedroom door open -ed this conclusion
的可能性有多大？

dekénéngxìng yǒu duōdà

‘sprobabilityhave how big

Suppose a person is sleeping in the living room and hears the sound of a door opening. What do you think is the likelihood of this person concluding that the bedroom door is open?

#### 3.1.3. Procedure

The experimental materials were divided into four lists in a Latin-square design, each containing 18 objective-bare sentences, 18 objective-marker sentences, 18 subjective-bare sentences, and 18 subjective-marker sentences. In addition, 18 fillers were added to each list. Each participant was randomly assigned to one of the four lists. Participants were asked, ‘How certain do you think the speaker is when he or she said that sentence?’, and instructed to answer on a scale of 1–6 (1 represents not certain at all and 6 represents completely certain).

### 3.2. Results

The mean rated speaker certainty was 4.39 (*SD* = 0.31) for objective-bare sentences, 4.04 (*SD* = 0.23) for objective-marker sentences, 4.48 (*SD* = 0.24) for subjective-bare sentences, and 4.09 (*SD* = 0.30) for subjective-marker sentences. The mean rated evidence strength was 62.62 (SD = 4.70) for objective sentences, and 58.38 (*SD* = 4.61) for subjective sentences. We found a significant three-way interaction among subjectiveness, marker, and evidence strength (*β* = 0.03, *SE* = 0.01, *t* = 2.23, *p* < 0.05, *CI.lower* = 0.00, *CI.higher* = 0.06). We looked at how marker interacts with evidence strength in subjective and objective statements to break down the three-way interaction. In subjective conditions, we found a main effect of evidence strength (*β* = 0.02, *SE* = 0.00, *t* = 3.67, *p* < 0.001, *CI.lower* = 0.01, *CI.higher* = 0.03). No matter whether the statement contained an evidential marker or not, participants’ perceived speaker’s certainty of subjective statements was higher as the evidence strength increased. In objective conditions, we found a marginally significant main effect of marker (*β* = −1.31, *SE* = 0.01, *t* = 1.45, *p* = 0.05, *CI.lower* = −2.60, *CI.higher* = −0.02). Participants perceived the speaker’s certainty to be lower if the statement contained a marker, regardless of how strong the provided evidence was. Linear mixed-effects models were fitted to the perceived certainty using the lme4 package version 1.1-35.1 (Bates et al., 2015) in R version 4.3.3 (R Core Team, 2024). We included subjectivity (sum-coded, subjective −0.5 vs. objective +0.5) and marker (sum-coded, bare −0.5 vs. marker +0.5), as evidence strength (numeric) as independent variables. Subjects were included as random intercepts. Any significant interactions were analyzed with the emmeans package version 1.10.0 (Lenth, 2024).

We found a significant three-way interaction among subjectivity, marker, and evidence strength (*β* = 0.03, *SE* = 0.01, *t* = 2.23, *p* < 0.05). We looked at how marker interacts with evidence strength in subjective and objective statements to break down the three-way interaction. In subjective conditions, we found a main effect of evidence strength (*β* = 0.02, *SE* = 0.00, *t* = 3.67, *p* < 0.001). No matter whether the statement contained an evidential marker or not, participants’ perceived speaker’s certainty of subjective statements was higher as the evidence strength increased. In objective conditions, we found a marginally significant main effect of marker (*β* = −1.31, *SE* = 0.01, *t* = 1.45, *p* = 0.05). We find a weak evidence effect in objective-bare sentences (*t Statistic* = 2.92, *Two-Tailed Critical Value* = 2.57, *p* < 0.05), but a significant main effect of marker in objective-marked sentences (*t Statistic* = −5.80, *Two-Tailed Critical Value* = 2.57, *p* < 0.01). Participants perceived the speaker’s certainty to be lower if the statement contained a marker, regardless of how strong the provided evidence was.

### 3.3. Discussion

The result suggests that evidence strength plays a role in subjective evaluations but not in objective sentences. In subjective evaluations, there is a perceived increase in the speaker’s certainty in direct proportion to the strength of the evidence presented. In objective sentences, evidence strength is not found to correlate with the speaker’s certainty, and an increase in the degree of evidence strength does not result in a corresponding increase in the perceived certainty of the speaker. We will discuss the results further in the General Discussion section.

## 4. Discussion

The present study sets out to see the impacts of the subjectivity of the sentence, the inferential markers, and the strength of the evidence on the perceived speaker’s certainty.

In Experiment 1, we found a significant interaction between the subjectivity of statements and evidential markers. Whereas bare objective statements were rated with a higher speaker certainty than bare subjective evaluations, no significant difference was found when they were used with an evidential marker. In Experiment 2, we found a significant correlation between evidence strength and the perceived speaker’s certainty in subjective evaluations whether inferential markers were presented, and a significant correlation between inferential markers and the perceived speaker’s certainty regardless of the evidence strength in objective sentences.

### 4.1. Effect of Evidential Markers on the Perceived Speaker Certainty

We found that evidential markers significantly changed the perceived degree of the speaker’s certainty in subjective and objective sentences. Regardless of the subjectivity of the statement, participants rated the sentences with evidential markers as encoding a lower degree of the speaker’s certainty. The results suggest that inferential evidential markers denote a certain degree of speaker commitment/certainty, at least in the Chinese language. Our results partially support the view that the information source and speaker commitment are inseparable (e.g., Fang, 2006; Du, 2015). However, the core evidence of the disjunction view (e.g., Gan & Yu, 2024; Yue, 2014) is about reported evidential markers in Chinese, which is not within our scope. The supporting sentences in which *jushuo* (reportedly) merely indicates the information source without reflecting the speaker’s certainty, as identified by Gan and Yu (2024), need to be empirically tested through experimental studies.
(16)举例来说，六十四卦的第一卦，乾卦，据说是

jǔlì láishuō liùshísìguàdedìyīguà qiánguà jùshuōshì

for instance 64hexagram‘sfirst hexagramQian hexagram reportedlybe

刚健之象；第二卦，坤卦，是柔顺之象。

gāngjiàn zhīxiàng dì‘érguà kunguàshìróushùnzhī xiàng

strength and vigor of image second Kun hexagrambegentleofimage

凡是满足刚健条件的事物，都可以代入有乾卦
fánshì mănzú gāngjiàntiáojiàndeshìwù dōukěyǐ dàirù yŏu qián’guà
every meetstrength and vigor condition ‘s thing all may bring into Qián
卦象出现 的公式里；凡是满足柔顺条件的事物，

guàxiàng chūxiàn de gōngshì lĭ fánshìmănzú róushùntiáojiàn de shìwù

hexagram appear ‘s formula in every meetdocility condition ‘s thing

都可以代入 有坤卦卦象出现的公式里。

dōu kěyǐdàirùyŏu kūn’guàguàxiàng chūxiànde gōngshìlĭ

all may bring into have Kun hexagram hexagram appear‘s formula in


For example, the first of the 64 hexagrams is the Qian hexagram, which is said to represent strength and vigor; the second hexagram, the Kun hexagram, represents docility and compliance. Any entity that meets the criteria of strength and vigor can be substituted into the formula where the Qian hexagram appears; any entity that meets the criteria of docility and compliance can be substituted into the formula where the Kun hexagram appears.

According to Gan and Yu (2024), *jushuo* in Example (16) is argued to be omissible as it seems to ‘appear at the same position in the following text. Here, it appears as a word to indicate the source of information for the sake of information integrity, and does not indicate the speaker’s understanding of the credibility of the information’, and, thus, it has no epistemic values. It seems that whether the Qian hexagram signifies strength and vitality lies entirely beyond the speaker’s knowledge, implying no epistemic value. In our view, without experimental support, it remains questionable whether the use of *jushuo* in Example (16) influences the speaker’s certainty.

Further experiments are needed to explore Chinese native speakers’ intuitions regarding the impact of reported markers, such as *jushuo*, on the perceived speaker certainty. The follow-up experiments may focus on the reported evidential markers to further clarify the relationship between evidentiality and epistemic modality in Chinese.

This study contributes to the existing body of literature on evidentiality by including subjective sentences, an element notably absent in the majority of the previous research. A large number of previous studies on evidentiality used factual/objective sentences because, in languages with grammaticalized evidential markers, subjective sentences are required to include an evidential marker (Tosun & Vaid, 2018). It is, therefore, not possible to create a minimal pair of subjective sentences in these languages. Chinese, using lexical evidential markers, allows for subjective sentences to be used both with and without evidential markers. Using Chinese as the stimuli, we provided evidence that supports the previous findings regarding the role of evidential markers in affecting the perceived speaker’s certainty (Tosun & Vaid, 2018; Arslan, 2020; Smirnova, 2021; Fishman & Degen, 2023; Teigen & Juanchich, 2024).

### 4.2. Modulation of Subjectivity of Statement on the Effect of Evidential Markers

In Experiment 1, we found a significant interaction between marker and subjectivity, with a significantly smaller effect of marker in subjective sentences than in objective sentences. The objective-marker sentences and subjective-marker sentences showed no significant differences, but subjective-bare sentences were rated much lower than objective-bare sentences. In other words, we found that adding an inferential marker leads to a greater reduction in the degree of the perceived speaker’s certainty in objective sentences than in subjective evaluation.

In an objective sentence, even without any evidential marker, the objective situation described is predominantly perception-based without much reasoning. Perceptual evidence is not difficult to find and is not speaker-oriented. In subjective evaluations, concrete perceptual evidence and profound reasoning are also required. The hearer cannot be fully certain about the speaker’s evaluation, in the absence of tangible perceptual evidence provided in the context. Based on limited knowledge in the context, the speaker is often not able to give a precise result, and, thus, the certainty perceived is not as strong as in objective sentences, as in Example (17).
(17) Contexts without evidence:你和张三被困在战壕中， 商量突围时间，nĭhéZhāng Sānbèikùnzàizhànháozhōng, shāngliáng tūwéishījiān,youandZhang San-edtrapstaytrench middle discuss breakouttimeYouandZhang Sanare trapped in the trench, discussing about the breakout time.张三对你说:“这个时间非常适合。”

Zhāng Sānduìnǐshuōzhègèshíjiānfēicánghéshì

Zhang Santoyousaythistimeverysuitable

Zhang San says to you: This time is very suitable.

In (17), ‘whether this time is very suitable’ can only be verified in the future, but is not verifiable at the speech time. The speaker’s certainty in subjective evaluations is not guaranteed, due to either a scarcity of perceptual information or a deficiency in professional knowledge. In contrast, objective sentences depict past and present situations that are easily perceivable, leading to an assessment where the speaker is perceived as possessing a higher degree of certainty.

By adding an inferential marker, objective sentences and subjective evaluations are changed into the result of inference. The inference of an objective situation indicates a lack of direct perceptual evidence, whereas the inference of one’s own subjective evaluation reports the speaker’s own feeling, which sounds more certain than inference about objective situations. Moreover, the initial position of the inferential marker takes a dominant role in deciding the certainty of speakers in Experiment 1 without evidence for a cause–effect relationship.

It remains an open question whether the current results can be replicated in other languages. While we assume that a subjective versus objective distinction still exists, the interaction between subjective sentences and evidential markers may result in different results in another language.

### 4.3. Interaction Among Subjectivity, Evidential Markers, and Evidence Strength

We found, in Experiment 2, that, in subjective evaluations, evidence strength is a key factor in determining the speaker’s degree of certainty, whereas, in objective statements, participants seem to assess the degree of certainty based on the markers, rather than inference based on evidence through their own reasoning. In objective sentences, the judgments of the speaker’s certainty are based on the language employed, without independently reasoning through the evidence presented. The perception of objective situations is assumed to be consistent among different conceptualizers; for instance, the statement ‘it is raining’ is not speaker-oriented, and any speaker should be able to judge whether it is raining.

**Figure 3:**
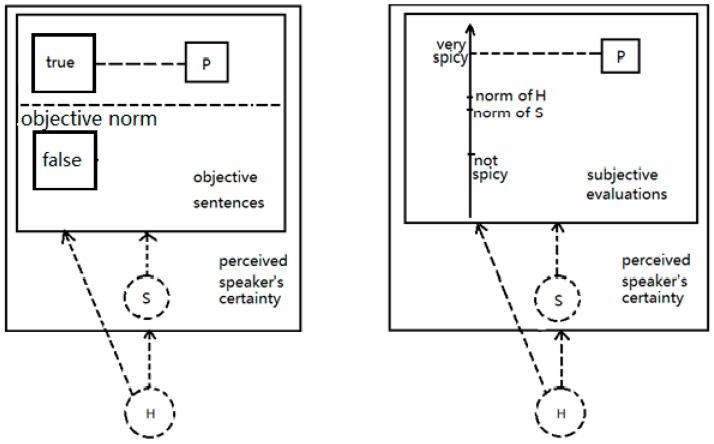
The perception of speech events in objective sentences and subjective evaluations (P: proposition; S: speaker; H: hearer).

Our stimuli consistently depicted scenarios with a close relationship between the speaker and the hearer (i.e., participants), e.g., couples, parent–child, friends, etc., which fostered a high level of trust. In objective situations, the close relationship between the speaker and the hearer leads to the hearer’s belief in the speaker. The closer the relationship, the greater the trust the hearer places in the speaker. Such a belief overrides the presented evidence, where the hearer has firm faith in the speaker’s ability to accurately interpret the situation, and there is no reason to suspect the speaker would be dishonest. The hearer trusts that the speaker possesses the knowledge to accurately describe objective situations, as the standard for such situations is not speaker-dependent, as depicted in the left panel of Figure 3. The evidence strength does not affect the speaker’s certainty perceived, as the hearer has faith in the speaker.

Conversely, in subjective evaluations, the participants bear in mind that evaluations vary among different people; for example, ‘the dish is very spicy’ may be true for people in Guangdong province but not for those in Sichuan province. The hearer tends to make his evaluation rather than taking the speaker’s evaluation for granted. The norm of the subjective situation is speaker-dependent, as shown in the right panel of Figure 3. In this case, hearers will need extra evidence to evaluate the situation as well as the speaker’s perception of the situation. Thus, in subjective sentences, participants’ judgment of the speaker’s certainty is affected by the strength of the evidence provided in the context.

If the speaker is a stranger, the results of both experiments may show significant differences. Contexts, either linguistic contexts or non-linguistic contexts, play a key role in interpreting the speaker’s certainty or commitment. In our study, we provide support for the evidence strength in the context influencing the interpretation evidential markers and certainty perception. Various other contextual elements may also impact the interpretation of evidence strength and evidential markers, such as the position of the linguistic element, prosodic cues, and cultural background (as mentioned in Yuan & Lyu, 2022), which are worthy of further examination.

A higher evidence strength is conventionally associated with an increased trust and certainty in scientific findings. However, this relationship may be undermined in public perception, as stronger evidence can paradoxically lead to lower trust, exemplified by the weak evidence effect identified by Fernbach et al. (2011).

The weak evidence effect is found in objective-bare sentences. In objective-bare sentences without evidence, participants rely solely on the speaker’s description and tend to believe in the speaker’s judgment. Objective-bare sentences without markers and evidence tend to be deemed as reporting objective situations and very reliable (certainty). In objective-bare sentences with evidence, participants seem to rely on the speaker’s description and also make a judgment based on the evidence. When the evidence is not strong enough leading to the result, a weak evidence effect is reached.

However, in both objective-marked sentences with and without evidence in the context, the marked sentences are deemed to report the result of the speaker’s inference, where evidence strength has a positive correlation with the perceived speaker’s certainty.

### 4.4. Limitations and Future Directions

One limitation of the present study is we did not distinguish the six evidential markers. Among the six markers adopted, *renwei* and *yiwei* seem to be somewhat different with the other four. The most frequent inferential marker, *wo renwei*, very much like ‘I think’ in English, has grammaticalized into a discourse marker, making it not simply an inferential marker. On the other hand, *wo yiwei* may include a negative attitude of the addresser (Xu, 2014), which may have an impact on the perceived speaker’s certainty. Another limitation, as mentioned in the discussion, is the materials used, where the speaker and the hearer dominantly have a close relationship.

Future studies can consider the positional flexibility of lexical evidential markers. Unlike grammatical markers, which often have fixed positions, lexical evidential markers can appear in various syntactic positions within a sentence. For example, *wo renwei* can be at the initial, the middle, the final, and the parenthetical position in the sentence as shown in (18), which are argued to perform different pragmatic functions (Li, 2016).
(18) a. 我认为我高攀了你们家。
I think I have climbed high in your family.
b. 最后这一推论相当合理, 我认为。
This conclusion seems quite reasonable, I think.
c. 回顾三年来的工作, 我认为, 文艺界是很有成绩的部门之一。
Looking back on the work of the past three years, I think, the cultural and artistic circles have been one of the most successful sectors.

Based on our assumption, the impact of evidential markers on the perceived speaker certainty may vary depending on their syntactic position. From the perspective of Cognitive Grammar, markers placed at the beginning of a sentence receive focal prominence and are likely to exert a more pronounced influence on perceived certainty. This suggests that the role of the syntactic position in shaping the perceived certainty conveyed by evidential markers is a factor that merits further empirical investigation.

Another important consideration is the degree of subjectivity, which can vary significantly. The sentences used in our experiments were designed to be either highly objective or highly subjective. However, in real-world contexts, many sentences fall somewhere in between these extremes. For instance, consider the sentence “Zhang San studies hard,” which is based on a social norm of diligence and reflects the speaker’s personal evaluation. This suggests that future research could focus on exploring the interaction between evidential markers and sentences that exhibit a moderate degree of subjectivity.

Future research could explore cross-linguistic perspectives, as suggested by the reviewers. Translational equivalents such as *we renwei* and *I think* can be distinguished by the degree of speaker certainty they convey. Moreover, it would be valuable to examine whether inferential markers as a whole category in languages without grammaticalized evidential markers, such as English, also serve to lower the perceived certainty of the speaker. The findings of this paper should be tested for their universality across different languages.

Another potential research direction is a comparison among different genres. While inferential markers are predominantly found in oral communication, they may be used in other contexts, such as professional or institutional contexts, as suggested by the reviewers. The validity of our findings requires verification in these contexts. Additionally, comparing these markers across different genres can enhance our understanding of their meanings and uses. Such comparisons could be informed by similar studies on casual connectives from various domains, as seen in the works of Andersson and Sundberg (2021) and Xiao et al. (2021). Similarly to comparing connectives across domains, comparing inferential markers across genres can shed light on how these linguistic elements function in different communicative settings.

Future studies might explore several additional potential variables. These include the positions of evidential markers (whether sentence-initial, medial, final, or parenthetical) in contrast to the fixed positions of grammatical evidential markers, the relationship between the speaker and hearer, and the intricacies specific to the Chinese language. Additionally, the inferential markers adopted seem to have varying degrees of influence on the speaker’s certainty, and, thus, future research may focus on elucidating these differences.

## 5. Conclusions

We conducted two experiments exploring the effects of inferential evidential markers, subjectivity, and evidence strength on the perceived speaker’s certainty. In Experiment 1, participants were asked to judge the perceived speaker’s certainty with and without an inferential marker in a context free of evidence. The results revealed that subjective evaluations are conceived with a lower degree of the speaker’s certainty than objective sentences, and Chinese evidential markers change the perceived speaker’s certainty in subjective and objective sentences. In Experiment 2, participants were asked to judge the strength of the cause–effect relationship between the evidence in the context and the result of the inference. Some other participants were asked to judge the perceived speaker’s certainty in the same context. The result suggested evidence strength played a role in subjective evaluations but not in objective sentences.

## Data Availability

The original contributions presented in this study are included in the article/Supplementary Material. Further inquiries can be directed to the corresponding author(s).

## Supplementary Materials

The following supporting information can be downloaded at: https://www.mdpi.com/article/10.3390/bs15070912/s1, File S1: Materials used in the experiment.

## Appendix A. The 18 Bare Objective Sentences and Their Evidential Forms Adopted in Experiment 1

	**Objective**		**Subjective**	
	**Bare**	**With Marker**	**Bare**	**With Marker**
1	红灯亮了。 The red light is on.	我**认为**红灯亮了。 I **think** the red light is on.	那个孩子非常聪明。 That child is very clever.	我**认为**那个孩子非常聪明。 I **think** that child is very clever.
2	米饭烧糊了。 The rice was burned.	我**认为**米饭烧糊了。 I **think** the rice was burnt.	这个学生非常认真。 This student is very careful.	我**认为**这个学生非常认真。 I **think** this student is very careful.
3	外面下雨了。 It is raining outside.	我**认为**外面下雨了。 I **think** it is raining outside.	这个手机很好用。 This phone functions very well.	我**认为**这个手机很好用。 I **think** this phone functions very well.
4	何鹏进屋了。 He Peng entered the room.	我**以为**何鹏进屋了。 I **think** He Peng entered the room.	这个城市很大。 This city is very big.	我**以为**这个城市很大。 I **think** this city is very big.
5	面包吃光了。 The bread has been eaten up.	我**以为**面包吃光了。 I **think** the bread has been eaten up.	那台电脑速度非常快。 That computer is very fast.	我**以为**那台电脑速度非常快。 I **think** that computer is very fast.
6	公众号更新了。 The public account (on WeChat) has been updated.	我**以为**公众号更新了。 I **think** the public account (on WeChat) has been updated.	这个动作非常难。 This movement is very difficult.	我**以为**这个动作非常难。 I **think** this movement is very difficult.
7	走廊灯坏了。 The corridor light is broken.	我**推测**走廊灯坏了。 I **infer** the corridor light is broken.	那个地方很冷。 That place is very cold.	我**推测**那个地方很冷。 I **infer** that place is very cold.
8	牛奶过期了。 The milk has expired.	我**推测**牛奶过期了。 I **infer** the milk has expired.	那个发型很好看。 That hairstyle looks very great.	我**推测**那个发型很好看。 I **infer** that hairstyle looks very great.
9	火车晚点了。 The train is late.	我**推测**火车晚点了。 I **infer** the train is late.	那个门非常坚固。 That door is very sturdy.	我**推测**那个门非常坚固。 I **infer** that door is very sturdy.
10	房门钥匙丢了。 The key is missing.	我**估计**房门钥匙丢了。 I **reckon** the key is missing.	那个游戏很好玩。 That game is very fun.	我**估计**那个游戏很好玩。 I **reckon** that game is very fun.
11	卧室门开了。 The bedroom’s door is open.	我**估计**卧室门开了。 I **reckon** the bedroom’s door is open.	这个瓶子很值钱。 This bottle is very valuable.	我**估计**这个瓶子很值钱。 I **reckon** this bottle is very valuable.
12	李飞毕业了。 Li Fei has graduated.	我**估计**李飞毕业了。 I **reckon** Li Fei has graduated.	这个菜很好吃。 This dish is very delicious.	我**估计**这个菜很好吃。 I **reckon** this dish is very delicious.
13	中国队赢了。 The Chinese team won.	我**猜（测）**中国队赢了。 I **guess** the Chinese team won.	那件衬衫很干净。 That shirt is very clean.	我**猜（测）**那件衬衫很干净。 I **guess** that shirt is very clean.
14	桥面结冰了。 The bridge has frozen over.	我**猜（测）**桥面结冰了。 I **guess** the bridge has frozen over.	这本书非常难。 This book is very difficult.	我**猜（测）**这本书非常难。 I **guess** this book is very difficult.
15	会议结束了。 The meeting has ended.	我**猜（测）**会议结束了。 I **guess** the meeting has ended.	那个菜非常辣。 That dish is very spicy.	我**猜（测）**那个菜非常辣。 I **guess** that dish is very spicy.
16	外面起风了。 It starts to be windy outside.	我**推断**外面起风了。 I **infer** it starts to be windy outside.	那辆车很便宜。 That car is very cheap.	我**推断**那辆车很便宜。 I **infer** that car is very cheap.
17	小区停电了。 There is a power outage in the community.	我**推断**小区停电了。 I **infer** there is a power outage in the community.	这个时间非常适合。 This time is very suitable.	我**推断**这个时间非常适合。 I **infer** this time is very suitable.
18	汽油涨价了。 The gasoline prices have gone up.	我**推断**汽油涨价了。 I **infer** gasoline prices have gone up.	这个人非常有趣。 This person is very interesting.	我**推断**这个人非常有趣。 I **infer** this person is very interesting.
